# AGREEMIP: The Analytical Greenness Assessment Tool for Molecularly Imprinted Polymers Synthesis

**DOI:** 10.1021/acssuschemeng.4c03874

**Published:** 2024-07-13

**Authors:** Mariusz Marć, Wojciech Wojnowski, Francisco Pena-Pereira, Marek Tobiszewski, Antonio Martín-Esteban

**Affiliations:** † Department of Analytical Chemistry, Faculty of Chemistry, Gdańsk University of Technology (GUT), ul. G. Narutowicza 11/12, 80-233 Gdańsk, Poland; ‡ Department of Chemistry, University of Oslo, P.O. Box 1033-Blindern, 0315 Oslo, Norway; § Centro de Investigación Mariña, Departamento de Química Analítica e alimentaria, Grupo QA2, Edificio CC Experimentais, Universidade de Vigo, Campus de Vigo, As Lagoas, Marcosende, 36310 Vigo, Spain; ∥ EcoTech Center, Gdańsk University of Technology (GUT), ul. G. Narutowicza 11/12, 80-233 Gdańsk, Poland; ⊥ Departamento de Medio Ambiente y Agronomía, INIA-CSIC, Carretera de A Coruña km 7.5, 28040 Madrid, Spain

**Keywords:** green metrics, green chemistry, green analytical chemistry, green sorbents, molecularly imprinted polymer synthesis

## Abstract

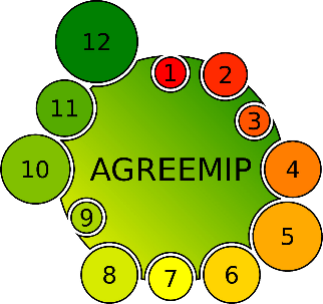

Molecular imprinting technology is well established in areas where a high selectivity is required, such as catalysis, sensing, and separations/sample preparation. However, according to the Principles of Green Chemistry, it is evident that the various steps required to obtain molecularly imprinted polymers (MIPs) are far from ideal. In this regard, greener alternatives to the synthesis of MIPs have been proposed in recent years. However, although it is intuitively possible to design new green MIPs, it would be desirable to have a quantitative measure of the environmental impact of the changes introduced for their synthesis. In this regard, this work proposes, for the first time, a metric tool and software (termed AGREEMIP) to assess and compare the greenness of MIP synthesis procedures. AGREEMIP is based on 12 assessment criteria that correspond to the greenness of different reaction mixture constituents, energy requirements, and the details of MIP synthesis procedures. The input data of the 12 criteria are transformed into individual scores on a 0–1 scale that in turn produce an overall score through the calculation of the weighted average. The assessment can be performed using user-friendly open-source software, freely downloadable from mostwiedzy.pl/agreemip. The assessment result is an easily interpretable pictogram and visually appealing, showing the performance in each of the criteria, the criteria weights, and overall performance in terms of greenness. The application of AGREEMIP is presented with selected case studies that show good discrimination power in the greenness assessment of MIP synthesis pathways.

## Abstract 2

A new metric tool and software that enables the assessment of the greenness of molecularly imprinted polymers synthesis procedures based on 12 criteria.

## Introduction

Molecular imprinting technology is well established in areas where a high selectivity is required, such as catalysis, sensing, and separations/sample preparation. This technology allows the synthesis of tailor-made polymers, called molecularly imprinted polymers (MIPs), that are capable of selectively recognizing a target compound (and its related compounds) from complex mixtures in rebinding experiments. The synthesis of MIPs involves a sequence of steps. First, a prepolymerization mixture containing a template molecule and suitable monomer(s) able to interact with each other in a proper solvent is prepared. The solvent plays a key role since it is responsible for stabilizing the template:monomer complex. Then, a cross-linker and initiator are added to the mixture, and polymerization is initiated either thermally or by UV radiation. After polymerization, the template is removed by exhaustive washing using a solvent capable of disrupting the template:monomer interactions. In this manner, the obtained polymer presents cavities complementary in size, shape, and functionality to the template molecule used during polymerization, thus providing a high degree of selectivity during rebinding assays. It is important to stress that the solvent (porogen) used also has a direct influence on the porosity of the resulting polymer, which in turn affects the diffusion of target analytes to the binding sites.^1−4^

Depending upon the final application, different polymerization strategies have been used (e.g., bulk polymerization, precipitation polymerization, etc.), which can be easily adapted to produce MIPs in different formats (beads, fibers) suitable for different sample preparation techniques such as solid-phase extraction (SPE), solid-phase microextraction (SPME), or stir bar sorptive extraction (SBSE), among others.^5,6^ Besides, MIPs can be combined with other materials such as carbon or quantum dots for the development of sensors,^7,8^ thus broadening the range of applications.

However, according to the Principles of Green Chemistry,^9^ the various steps required to obtain MIPs are far from ideal. First, most of the reagents used are toxic and, thus, pose a threat to both humans and the environment. Besides, depending on the polymerization route, a large amount of liquid or solid waste is produced, which represents an additional hazard and requires proper disposal. In this regard, some guidelines to make MIP synthesis greener have recently been proposed^10−12^ showing that each MIP synthesis step must be considered in order to reduce the operator’s exposure to toxic reagents, minimize energy consumption, and lower the amount of waste produced.

In recent years, the replacement of the typical acrylic-based monomers (i.e., acrylic acid and methacrylic acid), widely used for the synthesis of MIPs, has been the main area of research toward the obtainment of green MIPs. In particular, the use of natural polymers (biopolymers), such as chitosan, alginate, or cellulose, to prepare nontoxic, biodegradable, and biocompatible MIPs appears as a promising green alternative.^13,14^ In addition, such biopolymer-based MIPs can be easily adapted to current microextraction techniques.^15−17^ Similarly, the use of epoxidized soybean acrylate (ESOA), a vegetable oil-derived cross-linker, as an alternative to conventional cross-linkers was proposed for the synthesis of a biobased MIP, which showed high affinity and selectivity for resveratrol (template molecule).^18^ More recently, the use of silk fibroin for the preparation of MIP nanoparticles for the selective recognition of human serum albumin and hepcidin was proposed.^19,20^ Besides, the use of ionic liquids^21,22^ or deep eutectic solvents^23,24^ has been proposed and successfully employed in the synthesis of MIPs toward a variety of analytes showing similar or even superior performance compared to MIPs synthesized using conventional solvents (i.e., acetonitrile, toluene). It is evident from the above-mentioned works that greener alternatives to the synthesis of MIPs are already available, and thus, further research in this field is expected in the coming years.

However, although it is intuitively possible to design new green MIPs, it would be desirable to have a quantitative measure of the environmental impact of the different changes introduced for their synthesis. To the best of our knowledge, there is no dedicated tool for green assessment in the field of molecular imprinting, and in addition, the tools proposed for the assessment of the greenness of synthesis procedures of organic compounds or materials under laboratory conditions are not suitable for evaluating MIP synthesis. Among them, Eco-Scale and GREEN MOTION algorithms have been reported for evaluating the greenness of chemical reactions, based on subtracting a number of penalty points associated with different criteria from the ideal 100 value for an ideal reaction.^25,26^ However, a single overall score is obtained in the assessment; therefore, relevant specific information about the procedures (e.g., type of hazards) can go unnoticed. Alternatively, a metric tool with its associated software named Environmental Assessment Tool for Organic Synthesis (EATOS) enables the evaluation of the environmental impact of waste in synthetic procedures,^27^ although involving more laborious data collection. Life cycle assessment (LCA) has also been reported for the greenness assessment of chemical syntheses from a comprehensive multicriteria point of view.^28^ Unfortunately, LCA is data intensive and requires a large number of data collection times. Furthermore, the unavailability of relevant data is more common than desirable and can compromise the assessment. Data acquisition constraints have been overcome by combining LCA with previously reported green chemistry metrics,^29^ although without leading to substantial improvements in terms of assessment time.

Different metric tools have also been proposed for the greenness assessment of analytical methodologies and are applicable to methods involving MIPs for analytical purposes. National Environmental Methods Index (NEMI)^30^ and the Analytical Eco-Scale^31^ represented a starting point for the development of more refined metric tools, such as the Green Analytical Procedure Index (GAPI)^32^ and the Analytical GREEnness Metric approach (AGREE),^33^ the more commonly employed tools for the assessment of analytical methods. An improved version of GAPI, named complex-GAPI,^34^ has also been recently developed, including relevant criteria related not only to the analytical process being assessed but also to the processes performed prior to the analysis (e.g., synthesis of materials used in extraction). The introduction of more specific rather than general metric tools can effectively help in the evaluation of highly relevant steps of the analytical process. In this sense, the analytical method greenness score (AMGS) calculator^35^ has been developed for environmentally friendly chromatographic separations, whereas the introduction of the ten principles of green sample preparation^36^ and the necessity to evaluate sample preparation approaches, while considering their greenness profile with increased discriminatory capability over general analytical metric tools, led to the introduction of AGREEprep.^37,38^ These specific metric tools are particularly helpful in characterizing, comparing, and identifying preferable alternatives in terms of greenness without other stages of the analytical process influencing the assessment.

As far as we are aware, a limited number of contributions involving MIP synthesis have been assessed from the point of view of green (analytical) chemistry,^39−41^ even though such assessments necessarily overlooked the importance of the synthetic routes on the overall greenness of the method due to the lack of relevant MIP-specific criteria. Thus, the metric tools employed in these cases did not consider MIPs synthesis or did so in a superficial manner.

Thus, there is a clear need to develop a metric tool that can assist in the development and selection of greener routes for the preparation of MIPs. In particular, the metric tool should show an excellent ability to discriminate between synthesis routes of MIPs in terms of greenness while considering particularly relevant aspects of MIP procedures, guaranteeing a reasonable evaluation time, being straightforward, and producing results that are easy to interpret. In addition, the development of dedicated open-source software for the evaluation of MIPs would potentially allow the tool to be widely disseminated, which could lead to substantial improvements in future MIPs synthetic procedures. In this work, we present the first metric tool and software devised for the greenness assessment of synthetic protocols for MIPs preparation.

## AGREEMIP Criteria and Algorithms

**Figure 1:**
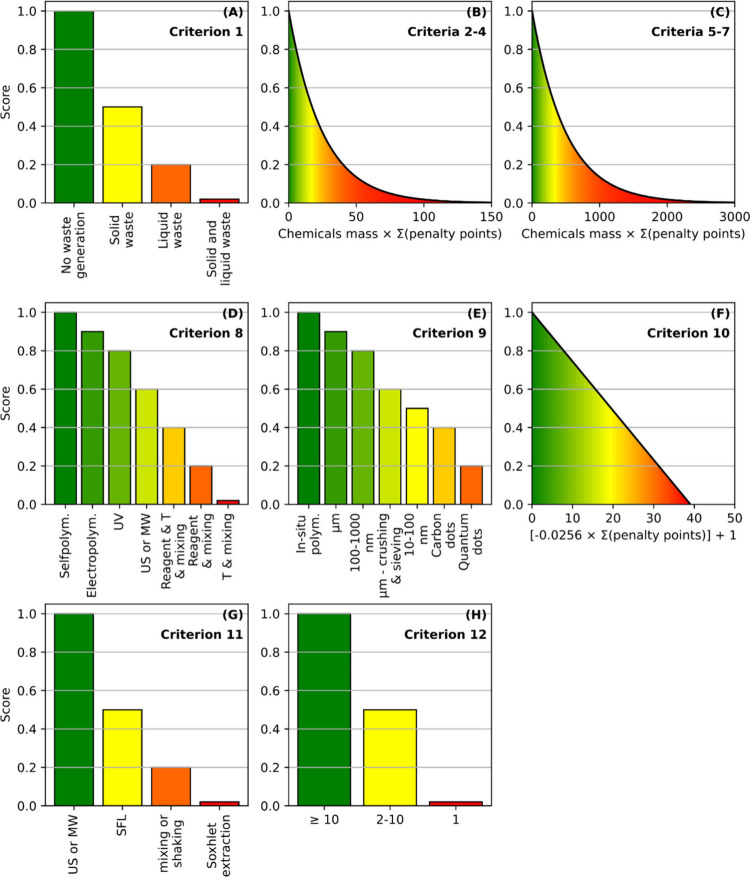
Graphical representation of the functions applied for the assessment of criterion 1 (A), criteria 2–4 (B), criteria 5–7 (C), criterion 8 (D), criterion 9 (E), criterion 10 (F), criterion 11 (G), and criterion 12 (H).

The assessment is based on an investigation of 12 criteria, transformation of every criterion into a standardized 0–1 scale and averaging, or weighted averaging, of the criterion scores into a single, overall score. After transformation, scores equal to 1 are assigned to the greenest conditions, while scores equal to 0 are given to the worst, nonacceptable situations. The functions used to transform the inputs for particular criteria into the standardized scores are criterion-specific to improve the resolution by mapping the entire 0–1 range to the reasonably expected range of inputs. In contrast, a stepwise transformation of raw data into scores is avoided whenever possible, as the resolution ability would be lost and is employed here only when the inputs are necessarily discrete/categorical in nature. These 12 criteria refer to different aspects of MIP synthesis and their summary and way of transformation follows. Figure 1 shows a graphical representation of the functions applied for the assessment of the 12 criteria.

### Criterion 1: Removal of Polymerization Inhibitors

The first criterion refers to the consequences of the removal of a polymerization inhibitor from polymerization mixture components. It can be done in several ways with solid or liquid waste generation or without any resulting waste. Of course, the best situation is when no waste is generated, in which case the score equals one. The same value is assigned if the components of the polymerization mixture are available free of the polymerization inhibitor. As a consequence, there is no need to perform this step, and the score of this criterion is one. However, in the case that there is a need to remove the inhibitor from the components of the polymerization mixture, the possibility of generation of solid waste is better (score = 0.5) than generation of liquid waste (score = 0.2) as handling of the former is easier. It can be separated by filtration, decantation, or centrifugation. The most problematic situation arises when solid and liquid waste are generated simultaneously, in which case the score is set to 0.

Since the composition of wastes is usually not known and is not stated in the scientific literature, this criterion does not deal with the generation of specific chemicals as wastes. Nevertheless, it should be noted that, in most cases, the inhibitor is removed once after the reagents are received. After this, if the purified reagents are stored under appropriate conditions, they might be used for the synthesis of polymers multiple times.

### Criteria 2–7: Functional Monomer, Template, Cross-Linking Agent, Porogen/Solvent and Other Reagents/Adjuvants/Carriers, Core/Particle Preparation, and Surface Modification

The chemicals comprising the polymerization mixture are treated in the same way but with every component relegated to a single criterion. Here, both the quantity of compound and the risk are considered. The mass of every component is multiplied by the sum of penalty points calculated based on hazard statements associated with them. The points assigned to each hazard statement are listed in Table S1 in the Supporting Information.^42^ In general, the more serious and unavoidable the effect, the higher the penalty and thus lower the criterion score.

Then, the product mass and hazards denoted in eq 1 as *x* is converted to a 0–1 scale with the following formulas:
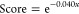
1
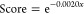
2where eq 1 is used to calculate the score in criteria 2–4 (functional monomer, template, and cross-linking agent, respectively) and eq 2 is used to calculate the score in criteria 5–7 (porogen, other reagents and core/particles preparation and surface modification, respectively).

The rounding of the score to two decimal points accounts for the boundary conditions. Equations 1 and 2 strongly favor nonhazardous reagents applied in small quantities. The increase in mass of reactant and/or more severe hazards results in a sharp lowering of the score, as can be observed in the graphical representation of the corresponding functions in Figure 1. Equations 1 and 2 are differentiated to produce better discrimination with inputs based on the reviewed papers.

It is important to point out that, in the case of mixtures of reagents (multimonomer, multitemplate, or solvent mixtures), for the simplicity of the AGREEMIP tool, the mass and hazards of each component are considered simultaneously.

### Criterion 8: Polymerization Initiation

The initiation of polymerization can be done in a few ways. The most traditional method involves the addition of a chemical agent (initiator), which is decomposed in free radicals by heating the mixture, thus triggering the polymerization reaction. While such a strategy is better than relying on just heating and/or mixing since it expedites the process, it is nonetheless inferior to some alternative approaches. Thus, just heating and mixing was assigned the lowest score of 0.0, mixing aided by the initiator, the score of 0.2, and the presumably most time- and thus energy-efficient combination of heating and mixing with the use of an initiator, the score of 0.4. Sonication or microwave-assisted initiation is considered to be more efficient in delivering energy to the reaction system; therefore, the score equals 0.6. A greener alternative is UV-mediated initiation which, although the addition of an initiator might be necessary, does not require excessive amounts of energy, and consequently, a score equal to 0.8 was given. Greener still are scenarios in which the polymerization initiator is not necessary, the polymerization reaction is fast, and the energy consumption is very low. For instance, such criteria are met for the synthesis of MIPs by electropolymerization, which was assigned a score of 0.9. Finally, the highest score of 1.0 was given to scenarios involving self-polymerization.

### Criterion 9: The Size of the Polymer Particles

The size of the polymer sorbent obtained is also related to the greenness of the system, as it affects how the material is disposed of after use and how the operator may be exposed to polymer particles of different sizes. The scores assigned to the different sorbent particle sizes are shown in Table 1. From our point of view, the greenest option, and therefore score 1, is to carry out *in situ* polymerization (i.e., preparation of imprinted monoliths to be used as fibers for SPME), as the entire polymerization mixture is used, thus avoiding the generation of waste. In addition, no further treatment (i.e., crushing and sieving) is required, and therefore, the operator is not exposed to fine particles. In this respect, the lower the particle size, the higher the risk of exposure to the operator, thus reducing the score. Accordingly, a score of 0.6 would be assigned to polymers prepared by bulk polymerization requiring crushing and sieving steps. In addition, the combination of MIPs with quantum dots and carbon dots, commonly used in sensor development, is considered the worst scenario due to the smallest size of particles used.

**Table 1:** Scores for Different Sizes of the MIP Particles

Size	Score
macroMIPs (extraction devices)	1
>1000 nm	0.9
1000–100 nm	0.8
μm (obtained after crushing and sieving)	0.6
10–100 nm	0.5
carbon dots	0.4
quantum dots	0.2

### Criterion 10: Template Elution Solvent

In order to obtain selective binding sites within the polymer network, the template must be removed by exhaustive washing using a proper solvent. Thus, the solvent used for template removal is an important criterion for the greenness assessment of MIP obtainment procedures. Since the amount of the solvent used is usually not stated in scientific papers and thus is generally not known, only the hazards associated with the solvent used are considered as penalty points according to Table S1. The function to recalculate points into a 0–1 scale is graphically represented in Figure 1 and shown below (eq 3) which, for penalty points higher than 39, produces a score equal to 0.
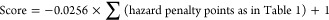
3

### Criterion 11: Template Elution Technique

The next criterion to be considered is the technique applicable to elute the template from the MIP. The least beneficial technique is the application of Soxhlet extraction, since it requires prolonged heating of the sorbent and lots of solvent, and thus, a score of 0 is assigned. Mixing or shaking usually requires lower solvent volumes and definitely lower energetic inputs; therefore, a score equal to 0.5 is given. Although not very common, it is also possible to use supercritical fluid extraction (SFE), which is considered a green technique since it mainly only uses CO_2_ in the supercritical state. However, since the experimental setup is complex (it requires the application of specialized equipment, which generates the high cost of both the equipment and the supercritical fluid), the score assigned is 0.8. The greenest option to remove the template is to stimulate the process with ultrasounds or microwaves, and here, a score of 1 is assigned.

### Criterion 12: Final Product Reusability

The last criterion refers to the application cycles of the MIP sorbent. The least favorable situation is when, after one sorption–desorption cycle, the sorbent has to be disposed of. For the single-use sorbent, the score equals 0. The second option is the application of MIP between 2 and 10 times, and then, the score equals 0.5. We do not take a more discriminative approach here, since in the literature, the information on sorbent reusability is scarcely more detailed than “more than once” or a “few times”. For 10 and more application cycles, the score equals 1 and it can be considered the most favorable condition.

### The Application of Weights

The weights are applied to differentiate the relative importance of the proposed criteria. The weight value may be indicated by the user on a four-point scale. A higher weight means a bigger importance when the weighted average is calculated. The default weights are suggestions based on expert opinion. They can, however, be modified according to users’ requirements or preferences. If the user decides to change the default weights, it is advised that the user justify the change. Detailed information about the suggested values of weights for defined criteria is listed in Table 2.

**Table 2:** Suggested Weights for the MIP Analysis

Criterion No.	Criterion Description	Weight
1	Removal of polymerization inhibitors	1
2	Functional monomer	2
3	Template	1
4	Cross-linking agent	3
5	Porogen/solvent	4
6	Other reagents, adjuvants, or carriers	3
7	Core/particles preparation and surface modification	2
8	Polymerization initiation	3
9	Size of polymer particles	1
10	Template elution solvent	4
11	Template elution technique	3
12	Final product reusability	3

The lowest weights are given to criterion 1, since the removal of the polymerization initiator is carried out once and the purified reagents can be used for many syntheses; criterion 3, since the amount of template molecules is smaller in the reaction mixture compared to the amounts of monomer, cross-linker, and porogen; and criterion 9, because the size of particles is to some extent related to environmental risks after the particles are released. Criteria 2 and 7 have been assigned weights equal to 2. In the case of criterion 2 this is because the amount of functional monomer in the polymerization mixture is larger than the template but smaller than the cross-linking agent and porogen while in the case of criterion 7 this is due to the fact that surface modification can have a significant impact on greenness. The highest weights are given to criteria 5 and 10, both referring to the application of large amounts of solvents as porogen and template elution solvent, respectively.

## AGREEMIP Software

A desktop application was developed to facilitate the use of the metric. It is based on the framework that was previously used for the AGREE and AGREEprep tools.^33,37^ It is open source and distributed freely under a MIT license. It was developed in Python 3.9 using the Tkinter module for graphical user interfaces.^43^ The source code can be obtained from an open repository maintained at git.pg.edu.pl/p174235/AGREEmip.

A standalone, compiled version is currently available for Windows. The most recent version is available at mostwiedzy.pl/AGREEmip. This is also where a link to a web-based version of the application with a similar functionality can be found.

It should be noted that, while the possible inputs cover most scenarios at the current state of the art, some cases might not be covered by any of the provided options, especially in the categorical criteria. In such cases, the users are asked to use their best judgment to select the closest equivalent and adjust the criterion weight if necessary.

## AGREEMIP Output/Pictogram

The main important final information is the final score of the assessed MIP synthesis procedure. To make the result more easily readable, the score is accompanied by a traffic light color scheme, with a red-yellow-green gradient mapped to the range from 0 to 1. The overall score in the middle of the pictogram gives information about the overall performance of MIP synthesis with the weights included. The information on the performance and weight of different criteria are visualized with smaller circles around the main circle, that also follow the color coding system. Easily readable information (color) on the criteria allows one to easily spot weak and strong points of the procedure and compare the performance of different procedures within the criteria. Another important piece of information carried by the result pictogram is what weights are applied, so what relative importance of the criteria was assumed. This is reflected by the size of the criteria circles around the central circle and their proximity to the center of the pictogram.

**Figure 2:**
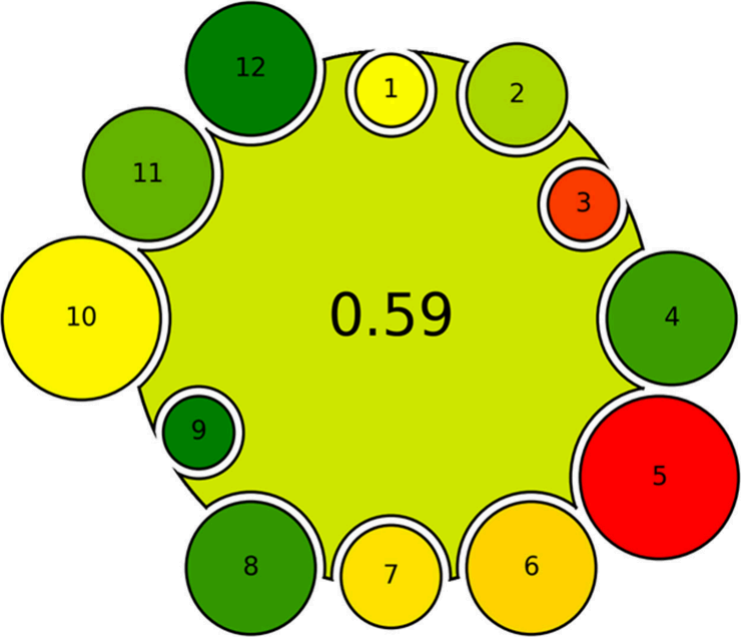
Example of the AGREEMIP assessment result, with the default weights applied.

The AGREEMIP pictogram refers to the MIP sorbent in its outlook, where the small circles represent the functional monomers interacting with the template: the large circle. In this manner, the pictogram can be immediately identified as the one dealing with MIP synthesis, differing from other metric tool pictograms devoted to the greenness assessment of analytical chemistry methods (i.e., AGREE or AGREEprep). The sample AGREEMIP result is shown in Figure 2.

## Case Studies

**Figure 3:**
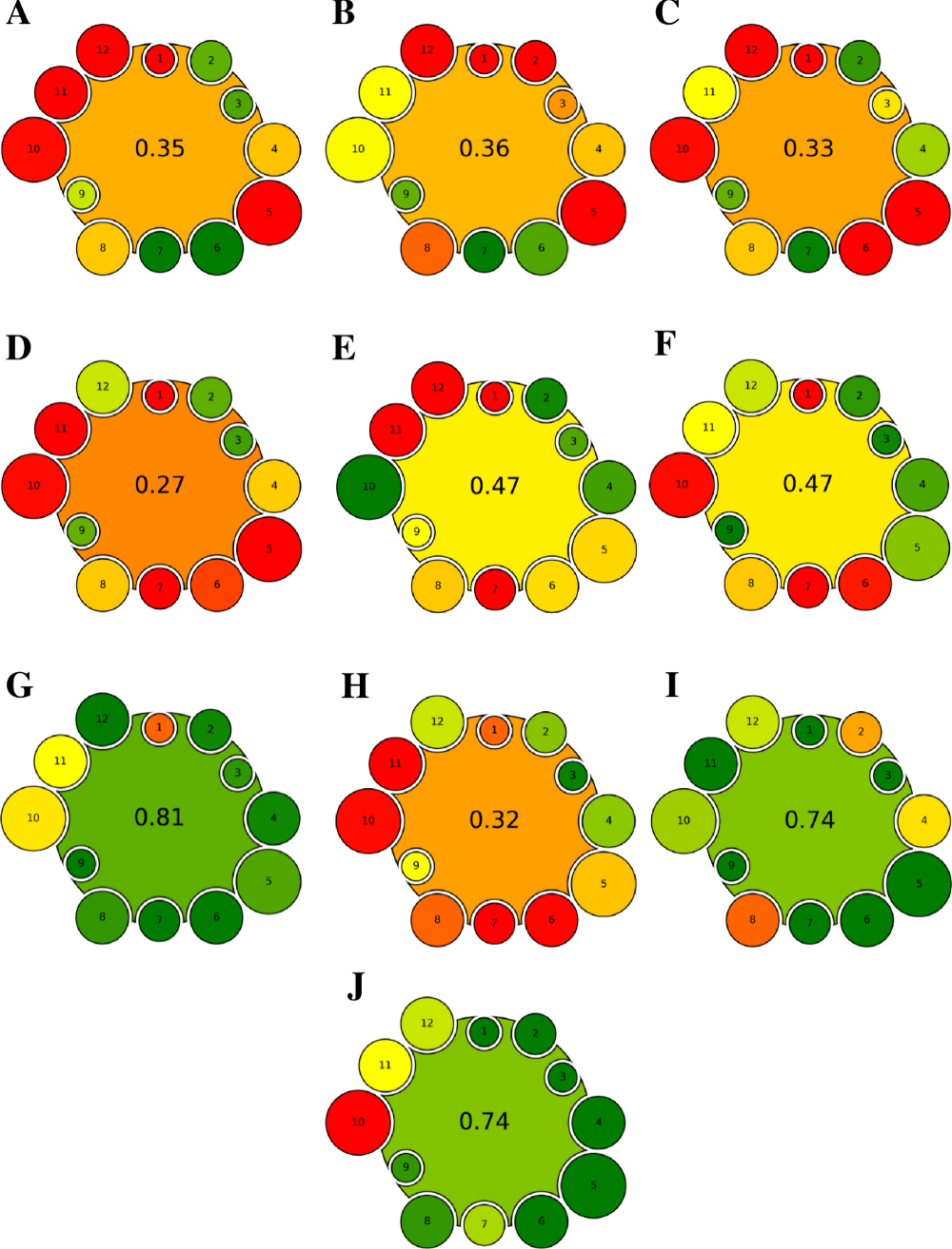
Results of AGREEMIP assessment of procedures for organic compounds determination: (A) MIP-SPE for quinalphos;^44^ (B) restricted access material-molecularly imprinted microspheres (including the addition of the comonomer);^45^ (C) mesoporous MIP core@shell hybrid silica nanoparticles;^46^ (D) dual-template surface MIP based on silica gel;^47^ (E) magnetic core–shell mesoporous MIP (Fe_3_O_4_–NH_2_);^48^ (F) MIP monolith containing magnetic nanoparticles for the SBSE;^49^ (G) electrochemically synthesized imprinted polymer for SPME;^50^ (H) magnetic dual-template MIP fabricated from a ternary biobased deep eutectic solvent (DES);^51^ (I) AngII-imprinted spongy columns (AngII-misc) synthesized for AngII detection from human serum;^52^ (J) flower-like sandwich-structured MIPs for efficient recognition of target protein from egg white.^53^

The field of applying MIPs in various types of configurations is constantly expanding. Therefore, in order to facilitate the demonstration of the usefulness of the developed tool, 10 different MIPs prepared following routes and used in analytical procedures for the determination of organic compounds in samples characterized by complex matrix composition were selected for case studies.^44−53^ The results of performed assessment studies using the AGREEMIP tool are shown in Figure 3.

The AGREEMIP scores on the ten investigated MIP preparation procedures range from 0.27 to 0.81. The highest scores for MIPs developed to measure xenobiotics in environmental samples were assessed for the G (0.81) and E and F (0.47) analytical procedures. In the case of the G analytical procedure,^50^ the synthesis and evaluation of imprinted films for SPME by electropolymerization of pyrrole alone or in the presence of ethylene glycol dimethacrylate (poly(pyrrole-*co*-EGDMA)-based MIP) was described. This analytical procedure scored highest due to the application of Cyclic Voltammetry for electropolymerization, and the use of just 4 mL of acetonitrile as a porogen was employed. In addition, the mass of the remaining components of the reaction mixture was significantly lower compared with other polymerization techniques. Moreover, during the electropolymerization process, the template (5 mmol of sulfadimethoxine) was entrapped during the formation of the polymer. A thin layer of MIP was deposited on the surface of the conductive electrode. In comparison to other polymerization techniques, the electropolymerization is significantly faster and less energy consuming and requires smaller amounts of reagents, and the template washing is carried out using a small volume of solvent.

As for the F analytical procedure, the preparation of monolith containing magnetic nanoparticles for the SBSE of thiabendazole and carbendazim from orange samples was characterized.^49^ The authors used magnetic nanoparticles that were surface modified with oleic acid and next encapsulated by a silica shell employing a conventional sol–gel procedure. For the preparation of the magnetic imprinted SBSE, the polymerization mixture and modified magnetic carrier were placed inside a glass vial insert for bulk polymerization: mixture consisted template, methacrylic acid (MAA), EGDMA, and 2,2′-azobis isobutyronitrile (AIBN) dissolved in a toluene:acetonitrile (ACN) 70:30 (v/v) mixture. Obtained monolith (SBSE MIP material) was washed to remove the template by the methanol:acetic acid (MeOH:HOAc) mixture. The use of a glass vial insert as a mold decisively minimizes the formation of unreacted components of the mixture, thereby reducing the possibility of generating additional waste.

A similar score (0.47) was assessed for the E polymerization procedure to obtain magnetic core–shell mesoporous MIP for selective recognition of triazoles residual levels in cucumber samples.^48^ The polymerization mixture consisted of magnetic nanoparticles (Fe_3_O_4_) as a MIP carrier and 3-aminopropyltriethoxysilane (APTES) and MAA as the functional monomer (several dozen of μL). Trimethylolpropane trimethacrylate (TRIM) was used as the cross-linking agent, and ACN was employed as porogen (10 mL). The polymerization reaction was performed by using chemical initiation (AIBN) under elevated temperature conditions (60 °C). The Soxhlet extraction was used to eliminate the residual template molecules.^48^

As for the I and J polymerization procedures, the green character (assessed result 0.74) is mostly associated with the application of water as the solvent during the synthesis of protein-imprinted polymers. Additionally, the protein templates can be removed from the polymer structure by water washing. In the case of the procedure described by Zhang et al.,^53^ NiO was employed as a material to immobilize ovalbumin, and dopamine was applied as a functional monomer. The most important is the fact that the entire preparation process was performed in an aqueous solution and without additional heating of the polymerization mixture. The problematic point in this case seems to be the use of acetic acid (20%, v/v) as one of the washing solutions (in addition to water and ethanol) to remove the template protein.

Yıldırım and Baydemir Peşint^52^ prepared AngII-imprinted spongy columns (AngII-misc) for the selective detection of AngII in human serum. As the general components of the polymerization mixture, 1-vinylimidazole, 2-hydroxyethyl methacrylate, *N*,*N*,*N*′,*N*′-tetramethyl ethylenediamine (TEMED), ammonium persulfate, and methylenebis acrylamide (MBAA) were used. To complete the polymerization process, the mixture with polymerization initiator was frozen in a cryostat at −16 °C for 10 h. To wash out the template molecule from the polymer structure, the desorption PBS buffer (10 mM, pH 7.4) and ultrapure water were used.

Furthermore, it might be noticed that the analytical procedure marked as D is characterized by the lowest total value of AGREEMIP assessment (0.27). Chen et al.^47^ describe polymerization conditions to obtain selective MIP material based on dual-template (imidacloprid and acetamiprid) and silica gel surface modification. Chemical compounds were mainly used during the synthesis of modified silica gel and the preparation of surface MIPs. Significant amounts of toluene were consumed in each of these steps (as a solvent and as a porogen agent). At the stage of synthesis of modified silica gel, HCl solution, pyridine, acryl chloride, and trimethylamine were used. Additionally, the obtained mixture was washed with toluene, acetone, ether, and methanol, which generates a significant amount of waste.^47^ It is evident that the use of such large amounts of solvents has a negative effect on the final score.

The scores of the remaining case studies (A–H) were quite similar, ranging from 0.32 to 0.36. As mentioned above, the score obtained should not be considered as an absolute value, and such small differences in the score value have no profound meaning. However, such scores highlight that procedures A, B, C, and H cannot be considered green and that some of the established criteria should be improved. In this respect, it is quite easy to identify areas for improvement just by looking at the pictograms shown in Figure 3, which demonstrate the usefulness and simplicity of the AGREEMIP tool.

## A Short Guide to What Should Be Reported in MIP Synthesis Publications

Considering the published papers associated with the MIPs preparation process, it might be found that there are some information gaps on the procedure conditions, reagent properties, and the amount of solvents used during synthesis. Currently, it seems impossible to include in a single tool such as AGREEMIP all of the relevant parameters related to the entire process of the desired MIP-type material synthesis. However, increasing the amount of data on this subject would allow us to make appropriate modifications in the future. In order to be able to make a better comparison of the procedures’ greenness, it is suggested that the data enclosed in scientific papers should include the following elements:

(i) Information about the method of purification of functional monomers and cross-linking agents (or other additional components) from inhibitors of the polymerization reaction. This will make it easier to define whether solid or liquid waste is generated, depending on the component purification technique used. In addition, this procedure is performed only once for a large volume of reagents and the postpurified reagent can be stored and used repeatedly.
(ii) Reusability of the developed imprinted material; this is one of the key elements that distinguishes molecularly imprinted sorbents from commercially available, routinely used, disposable solid sorbents that are the filling of, e.g., SPE columns. The more times it is possible to efficiently use the developed MIP in an analytical procedure, the less solid waste is generated. Research on imprinted sorption materials and publications should include such information on the potential multiplicity of the use of developed MIP-type sorbents without adversely affecting the ability to selectively bind target molecules.
(iii) In order to better illustrate the greenness of the entire procedure for obtaining new MIP sorbents, it is important to include information on the volume of solvent consumed during the process of washing the template from the polymer skeleton. Usually in the scientific publications, there is only information on the type of reagent used and the multiplicity of washing of the obtained material, but the key parameter from the point of view of assessing the greenness of the procedure, which is the volume of solvent consumed, is not included.
(iv) It would be beneficial to include information on the efficiency/effectiveness of the polymerization reaction, whether all components of the reaction mixture have completely reacted and no additional waste in the form of unreacted compounds has been generated.
(v) Information on whether the polymer sorbent support used (if any) was purchased as a finished product (e.g., silica gel or iron oxide) or had to be prepared independently under laboratory conditions. If this was necessary, it is also worth mentioning the efficiency of the reaction to obtain supporting particles, since additional waste may be generated at this stage as well.
(vi) In the sections describing the reagents and standards used, it would be very helpful to include information on the CAS No. of the given compound. It would definitely be easier for potential users of the AGREEMIP tool to find information on desired compounds, especially in terms of their physicochemical and toxic properties.

## Conclusions

A tool for easy assessment of MIP synthesis greenness has been developed. Up to now, such assessments were not easy to perform, but the proposed AGREEMIP tool, a user-friendly software, allows one to perform a simple and rapid assessment; the obtained result is readily interpretable. By random selection of some case studies, it has been demonstrated that the developed tool also shows good discrimination ability in greenness assessment, thus providing useful information to the user on which aspect of the synthesis should be modified to make the process greener. In this regard, it is important to stress that a given score is not to be considered as an absolute value but as a reference for the improvement of the greenness of the synthesis procedure. This is relevant considering the wide spectrum of polymerization techniques used for the synthesis of MIPs making the development of a quantitative universal assessment tool problematic. Besides, until now, in most of the published scientific papers, there is a lack of complete description of the experimental conditions used for the synthesis of MIPs, and thus, we encourage scientists working in this field to provide as much information as possible on the synthesis procedure used. In this manner, more sound AGREEMIP scores will be obtained, thus providing a more accurate picture of the greenness of the different MIP synthesis procedures.

## Supporting Information Available

Table S1, the penalty points assigned to the MSDS hazard statements based on ref (42) (PDF)
