# Supplementary material for: AGREEMIP: The Analytical Greenness Assessment Tool for Molecularly Imprinted Polymers Synthesis
Source: ACS Sustain Chem Eng. 2024 Jul 13;12(33):12516–24. doi: 10.1021/acssuschemeng.4c03874 (PMC11339656; doi:10.1021/acssuschemeng.4c03874)
Supplement: Supplementary file 1 — sc4c03874_si_001.pdf [file sc4c03874_si_001.pdf]

Supporting information for

AGREEMIP – the analytical greenness assessment tool for molecularly imprinted polymers synthesis

Mariusz Marć[1], Wojciech Wojnowski*[1,2], Francisco Pena-Pereira[3], Marek Tobiszewski[1,4], Antonio Martín-Esteban[5]

1 – Department of Analytical Chemistry, Faculty of Chemistry, Gdańsk University of Technology (GUT), ul. G. Narutowicza 11/12, 80-233 Gdańsk, Poland; wojciech.wojnowski@pg.edu.pl

2 – Department of Chemistry, University of Oslo, P.O. Box 1033-Blindern, 0315 Oslo, Norway

3 – Centro de Investigación Mariña, Departamento de Química Analítica e alimentaria, Grupo QA2, Edificio CC Experimentais, Universidade de Vigo, Campus de Vigo, As Lagoas, Marcosende, 36310 Vigo, Spain

4 – EcoTech Center, Gdańsk University of Technology (GUT), ul. G. Narutowicza 11/12, 80-233 Gdańsk, Poland

5 – Departamento de Medio Ambiente y Agronomía, INIA-CSIC, Carretera de A Coruña km 7.5, 28040 Madrid, Spain.

** wojciech.wojnowski@pg.edu.pl*

Number of pages: 3
Number of tables: 1
Number of figures: 0

Table S1. Transformation of hazards statements from MSDS into penalty points, based on earlier work. [1]

Hazard statement (H)	Description	Penalty points
H224	Extremely flammable liquid and vapour	9
H225	Highly flammable liquid and vapour	7
H226	Flammable liquid and vapour	5
H227	Combustible liquid	4
H228	Flammable solid	3
H242	Heating may cause a fire	3
H251	Self-heating; may catch fire	3
H261	In contact with water releases flammable gas	2
H270	May cause or intensify fire; oxidizer	7
H271	May cause fire or explosion; strong oxidizer	10
H272	May intensify fire; oxidizer	3
H280	Contains gas under pressure; may explode if heated	2
H290	May be corrosive to metals	2
H300	Fatal if swallowed	10
H301	Toxic if swallowed	7
H302	Harmful if swallowed	5
H304	May be fatal if swallowed and enters airways	8
H310	Fatal in contact with skin	10
H311	Toxic in contact with skin	7
H312	Harmful in contact with skin	5
H314	Causes severe skin burns and eye damage	5
H315	Causes skin irritation	2
H317	May cause an allergic skin reaction	2
H318	Causes serious eye damage	7
H319	Causes serious eye irritation	5
H330	Fatal if inhaled	10
H331	Toxic if inhaled	7
H332	Harmful if inhaled	5
H334	May cause allergy or asthma symptoms or breathing difficulties if inhaled	3
H335	May cause respiratory irritation	2
H336	May cause drowsiness or dizziness	2
H340	May cause genetic defects	7
H341	Suspected of causing genetic defects	7
H350	May cause cancer	10
H351	Suspected of causing cancer	7
H360	May damage fertility or the unborn child	10
H360d	May damage fertility	7
H360f	May damage the unborn child	7
H361	Suspected of damaging fertility or the unborn child	7
H361d	Suspected of damaging the unborn child	7
H361f	Suspected of damaging fertility	7
H362	May cause harm to breast-fed children	8
H370	Causes damage to organs	10
H371	May cause damage to organs	8
H372	Causes damage to organs through prolonged or repeated exposure	8
H373	May cause damage to organs through prolonged or repeated exposure	7
H400	Very toxic to aquatic life	10
H401	Toxic to aquatic life	8
H410	Very toxic to aquatic life with long-lasting effects	10
H411	Toxic to aquatic life with long-lasting effects	10
H412	Harmful to aquatic life with long-lasting effects	7
H413	May cause long-lasting harmful effects to aquatic life	5

Reference

(1) Tobiszewski, M.; Namieśnik, J.; Pena-Pereira, F. A Derivatisation Agent Selection Guide. *Green Chem.* **2017**, *19* (24), 5911–5922. https://doi.org/10.1039/C7GC03108D.
